# The Mediation Role of Emotion Regulation in the Relationship Between Anxiety and Depression in University Students with Specific Learning Disorder

**DOI:** 10.3390/healthcare13101211

**Published:** 2025-05-21

**Authors:** Michela Camia, Angela Ciaramidaro, Erika Benassi, Sara Giovagnoli, Damiano Angelini, Sandro Rubichi, Maristella Scorza

**Affiliations:** 1 Department of Biomedical, Metabolic and Neural Sciences, University of Modena and Reggio Emilia, 42121 Reggio Emilia, Italy; angela.ciaramidaro@unimore.it (A.C.); sandro.rubichi@unimore.it (S.R.); maristella.scorza@unimore.it (M.S.); 2 Department of Education and Humanities, University of Modena and Reggio Emilia, 42121 Reggio Emilia, Italy; erika.benassi@unimore.it; 3 Department of Psychology “Renzo Canestrari”, University of Bologna, 40127 Bologna, Italy; sara.giovagnoli@unibo.it; 4 Clinical Neuropsychology and Adult Dyslexia Unit, Neurology Department, Arcispedale S. Maria Nuova, 42123 Reggio Emilia, Italy; damiano.angelini@ausl.re.it

**Keywords:** emotion regulation, anxiety, depression, learning disabilities

## Abstract

**Background:** Difficulties in emotion regulation are associated with a range of emotional disorders, such as anxiety disorders and depression. However, the mechanisms that underpin this relationship are still unclear. Moreover, little is known about emotion regulation in university students with specific learning disorder (SLD). This study examined emotion regulation and its role as mediator in the relationship between anxiety and depressive symptoms in a group of university students with and without SLD. **Methods:** One hundred and twenty-nine Italian university students between 18 and 31 years of age were enrolled. Fifty students had a diagnosis of SLD and seventy-nine were typically developing students (TD). The data were obtained from a cross-sectional study conducted as part of a broader research project investigating the well-being of university students. Participants completed questionnaires for emotion regulation (DERS), anxiety (BAI), and depression (BDI). **Results:** In comparison to the group without SLD, the participants with SLD self-reported more frequent emotional regulation problems and more symptoms of anxiety and depression. SLD students also reported more difficulties in three domains of emotion regulation (nonacceptance, strategies, and clarity). Moreover, in both SLD and TD students, emotion regulation was found to mediate the association between anxiety and depression. **Conclusions:** The study suggests the importance of promoting adaptive emotion regulation strategies in university students with SLD and incorporating emotion regulation as a clinical target into existing interventions.

## 1. Introduction

Specific learning disorder (SLD) is a common neurodevelopmental disorder, with a prevalence ranging from 5 to 15% [1], characterized by persistent difficulties in the acquisition of reading, spelling, writing, and mathematics, despite adequate intelligence, intact sensory abilities, and appropriate instruction [1]. SLD is a lifelong condition and is often associated with difficulties in other cognitive domains. The clinical manifestations of these individuals can be highly variable and are sometimes referred to as both internalizing and externalizing disorders. Approximately thirty percent of children with SLD also have emotional and behavioral disorders [2].

Emotion regulation refers to the processes by which individuals modulate their affective experiences using cognitive, behavioral, interpersonal, and intrapersonal strategies [3]. Comprehensive conceptualizations of emotion regulation encompass multiple facets of the self-regulatory process, including emotion awareness, the acceptance of emotions, the ability to proceed with goal-directed actions when experiencing negative emotions, and the ability to flexibly apply emotion regulation strategies in order to meet individual goals and situational demands [4]. The absence of any or all of these abilities would indicate the presence of difficulties in emotion regulation, that is of emotion dysregulation [4]. Moreover, the relationship between emotion regulation strategies and gender has been investigated. In particular, Thayer [5] analyzed the complex relationship between gender, emotion regulation, and depression in young adults, and reported greater attention to emotions in women than men and that women think and ruminate more about their emotions compared to men; moreover, the comparison between women and men with high depressive symptoms revealed that women showed more impaired emotional strategies. Overall, the data reported that women seem to use more internally focused and passive responses, whereas men tend to use suppression and avoidance more frequently. In addition, women showed greater effortful control abilities and a greater tendency to use social support than men [6].

In the adult population, emotion regulation has been described as a transdiagnostic concept [7]. In fact, different mental disorders exhibit difficulties in emotion regulation, such as in patients with bipolar disorder [8], autism spectrum disorder [9], and attention deficit and hyperactivity disorder [10,11]. Furthermore, emotion regulation seems to play a mediation role in the maintenance of and the association between psychopathological symptoms. In the context of impulsive behaviors, emotion regulation difficulties mediated the relationship between anxiety and pathological gambling [12]. Similarly, Mansueto [13] found a mediating role of emotion regulation in the association between perfectionism and eating disorders.

Research has also emphasized that difficulties in emotion regulation are linked with a wide range of anxiety symptoms. For example, adults with generalized anxiety disorder showed maladaptive patterns of emotion regulation that are characterized by difficulties in understanding and regulating emotions. Thus, in patients with anxiety, emotions are quickly experienced and with high intensity [14]. The analyses of the strategies used in patients with social anxiety showed frequent use of maladaptive emotion regulation strategies, such as expressive suppression and rumination, compared to never-disordered controls [15].

Previous studies also have examined emotion regulation in depression. Research focused on the strategies used to respond to the affective states and to modify them. Depression seems to be associated with a frequent use of maladaptive strategies, such as rumination and suppression, and with a less frequent use of adaptive strategies, such as distraction and reappraisal [16].

Emotion regulation and its link to psychopathological symptoms in students has recently become a new concern for clinicians; in fact, data have shown a significant increase in mental problems in students during the last 15 years. Bamber and Kraenzle Schneider [17] reported that anxiety symptoms in college students have doubled from 2010 to 2015. In particular, the symptoms were higher in females than in males. Moreover, the presence of anxiety and depression seems to have an impact on academic achievements and seems to cause excessive stress in dealing with typical academic achievement, competition, and fear of failure. In particular, anxiety represents a maladaptive response to academic requests and consequently hampers intellectual functioning, such as memory, attention, and problem solving, causing a decrease in academic performance [17].

The persistence of maladaptive anxiety can also determine depression, rumination, avoidance, and psychosomatic disturbances in nursing students [18]. Malik and colleagues [19] found that, in university students, maladaptive emotion regulation and anxiety were positively associated. Thus, the way that students use emotion regulation plays an important role in their mental health and academic path [20].

The link between emotion regulation strategies, depression, and anxiety in youth has been analyzed and the results show that adaptive emotion strategies (cognitive reappraisal, problem solving, and acceptance) are negatively associated with psychological symptoms, whereas maladaptive strategies (avoidance, suppression, and rumination) are positively associated with depression and anxiety [21]. Interestingly, Dochnal [22] found that, independent of the presence or absence of comorbid anxiety, adolescents with depression used less adaptive regulation strategies. On the contrary, the presence of lifetime anxiety in comorbidity with depression was linked to the use of more maladaptive strategies.

Despite the fact that anxiety and depression frequently coexist, little is understood about the mechanisms behind this association. Different models have been proposed and, more recently, a number of studies used network analyses to investigate the link between anxiety and depression. Emotion regulation has been considered as one plausible construct that could explain the relationship between depression and anxiety symptoms.

Muris [23] proposed a theoretical model of the relationship between emotion regulation, anxiety, and depression in adolescents. The study focused on a specific emotion regulation process, namely behavioral inhibition, and the authors proposed a hypothetical model on the role of behavioral inhibition in the experience of anxiety and depression. According to the model, behavioral inhibition leads to anxiety, which, in turn, results in depression. This suggests that behavioral inhibition is a risk factor for later psychopathological symptoms. In particular, behavioral inhibition seems to have a direct effect on anxiety, which, in turn, leads to depression. Also, the negative form of emotion regulation (rumination, self-blame, and catastrophizing) is considered to be associated with depression and anxiety in adolescents and adults [24]. Moreover, negative emotion regulation has been described as a core feature in the maintenance of internalizing symptoms [25].

McGlinchey [26] conducted network analysis using data from a study on female adolescents between the ages of 11 and 18. Three structures were generated according to the level of emotion regulation (low emotion regulation, intermediate emotion regulation, and high emotion regulation). In the case of low and intermediate emotion regulation, the analysis found no clear separation between the symptoms of anxiety and depression. Thus, even in the case of different processes of emotion regulation, in adolescents, the two psychological constructs seem to be very closely linked.

Recently, Ruan and colleagues [27] explored the difficulties in emotion regulation in 209 adolescents in clinical settings. The authors used network analysis to verify the connection between emotion regulation, emotion reactivity, anxiety, and depression. They examined the six scales included in the Difficulties in Emotion Regulation Scale (DERS) and their relationships with the scores of the Hospital Anxiety and Depression Scale (HADS) assessing anxiety and depression. Among the different processes of emotion regulation, using limited strategies to regulate emotions was the most central node in the network. Moreover, anxiety symptoms were strongly associated with emotion reactivity, whereas depressive symptoms were more associated with difficulties in emotion regulation.

To the best of our knowledge research on emotion regulation in students with SLD is still debated. Parents of children with SLD reported higher levels of emotional dysregulation, anxiety, and physical symptoms compared to parents’ evaluations of typically developing peers (TD) [28]. In particular, children with SLD were described as having more symptoms of hyperactivity, including inattention, more emotional difficulties, and conduct problems. Özyurt and colleagues [29] found that adolescents with developmental dyslexia experienced greater problems in emotion regulation compared to their TD peers. The main difficulties were found in the following three areas: goal-directed behaviors, controlling impulsivity while experiencing emotions, and nonacceptance of emotions. Moreover, recent studies noted that children and adolescents with developmental dyslexia and ADHD had a poor understanding of emotions, difficulty in controlling their impulsivity, and fewer cognitive reappraisal strategies to regulate emotions [30]. It is also known that university students with dyslexia demonstrate problems with working memory, processing speed, planning, task monitoring, and organization, and these difficulties are perceived as affecting everyday activities [31,32]. Thus, having lower learning abilities could lead to lower self-efficacy, psychological problems, and lower levels of psychological well-being [33]. In particular, somatic complaints, social problems, lower self-esteem, more negative emotions, anxiety, sadness, and depression have been reported to be more consistent in the SLD population [34,35,36]. Still, with regard to anxiety and depression, some studies reported no differences between university students with SLD and TD students [37].

Given the evidence that students with SLD exhibit an elevated risk for anxiety and depressive symptomatology [36], and considering the established association between anxiety, depression, and emotion regulation [21,25,27], it is of clinical interest to further investigate the interplay between psychological symptoms and emotion regulation mechanism within this population. The transition to university represents a critical period of potential emotional vulnerability, especially for those with SLD [36], as increased academic demands and changes in daily life may negatively affect psychological well-being [38]. Therefore, emotion regulation may play a crucial role in promoting psychological adjustment, whereas dysfunctional emotion regulation processes could exacerbate the severity of psychopathological symptoms, such as anxiety and depression, which are especially relevant in students with SLD, given their increased vulnerability to emotional distress [39,40].

However, the current body of research on emotion regulation has primarily focused on clinical populations with established psychopathologies, such as individuals with gambling disorders or eating disorders [12,13]. Findings on those clinical groups have shown that emotion regulation can mediate the relationship between anxiety and other psychopathological disorders, largely neglecting the SLD population [41]. Building on these findings, we propose that emotion regulation may similarly mediate the relationship between anxiety and depression in students with SLD, highlighting the need for further investigations in this specific group.

Considering the aforementioned points, the main purpose of this study was to investigate emotion regulation, anxiety, and depression in SLD university students. We expected more difficulties in emotional regulation in students with SLD compared to TD students, as well as more anxiety and depression symptoms in the clinical group. A second aim was to identify specific regulatory domains that may be particularly affected in the SLD population. In line with previous research, differences were anticipated between students with and without SLD in aspects related to self-regulation and emotional processing.

Furthermore, the third goal was to analyze the association between emotion regulation, anxiety, and depression, and we hypothesized an association between these three psychological variables [15,22,23]. Specifically, we examined whether emotion regulation could contribute to the association between anxiety and depression. To our knowledge, no prior studies have focused on the possible mediating role of emotion regulation in the association between anxiety and depression in university students with and without SLD. However, in line with the previous literature that reported the role of emotion regulation in the association between different psychopathology symptoms [13], we hypothesized that emotion regulation mediates the association between anxiety and depression.

## 2. Methods

### 2.1. Participants

The study involved 129 university students between 18 and 31 years of age (mean age = 21.51; SD = 2.51) (see Table 1). The sample included 50 students with a diagnosis of SLD (mean age = 21.10, SD = 2.82; 42% male) and 79 TD students (mean age = 21.77; SD = 2.28; 43% male). The independent-samples *t*-test indicated that the age variable was not significantly different between the two groups (t_(127)_ = 1.487, *p* = 0.404, d = 0.261).

The diagnosis of SLD was based on the criteria included in the ICD-10 coding system (World Health Organization) [42] and conformed to the norms reported in the National Italian Consensus Conference on SLD published by the Italian Ministry of Health [43]. TD and SLD students with visual or hearing impairments and neurological or psychiatric problems were excluded from the study. All of the participants were Italian native speakers.

Data were collected as part of a cross-sectional study carried out in Northern Italy between January and July 2024, within the framework of a larger research project examining the well-being of university students. Participants with SLD were recruited from clinical centers specializing in SLD and neurodevelopmental disorders. TD students were recruited via notices posted at the university and via the university mailing list. The students interested in participating in the study were provided with a detailed explanation of the aims of the study, the voluntary nature of their participation, and their right to withdraw from the study at any time. Following the signing of the informed consent form for participation in the study, data analysis, and data publication, participants who agreed to take part in the study were provided with a link to the Google Forms platform to complete the questionnaires.

The study met the ethical guidelines for human subject protection, including adherence to the legal requirements of the country (Declaration of Helsinki), and it received formal approval from the local research Ethical Committee (protocol code 2023/0044335).

### 2.2. Self-Report Measures

The *Difficulty in Emotion Regulation Scale* (DERS) [44] is a widely-used self-report measure assessing the ability to regulate intense and negative emotions. The DERS includes 36 items which are rated using a 5-point Likert scale (1 = “*almost never*” to 5 = “*almost always*”). Higher scores reflect greater emotion regulation difficulties. The DERS includes the following six subscales: nonacceptance (investigating the tendency to respond negatively or with nonacceptance to one’s own distress (Cronbach’s alpha of 0.88)), goals (evaluating the challenges in focusing and completing tasks while experiencing negative emotions (Cronbach’s alpha of 0.85)), impulse (referring to the difficulty of maintaining control over one’s behavior during negative emotional experiences (Cronbach’s alpha of 0.5)), emotional awareness (indicating a lack of awareness or disregard for emotional responses (Cronbach’s alpha of 0.83)), strategies (investigating the belief that there is a limited ability to self-regulate once upset (Cronbach’s alpha of 0.81)), and clarity (evaluating the degree to which a person understands and is clear about his/her emotions (Cronbach’s alpha of 0.74)). The DERS total score has been shown to possess good internal consistency (Cronbach’s alpha of 0.90) [44].

The *Beck Anxiety Inventory* (BAI) [45,46] is a self-report measure including 21 items that assess the main components of anxiety, such as “Numbness or tingling”, “Feeling hot”, and “Dizzy or lightheaded”. The items are rated on a 4-point Likert scale (0 = “Not at all” and 3 = “Severe”). Higher scores indicate higher levels of anxiety. The BAI has been shown to possess good psychometric properties across clinical and community samples [Sica] (Cronbach’s alpha of 0.89).

The *Beck Depression Inventory* (BDI) [47,48] is a self-report measure assessing symptoms of depression. It includes 21 items that are rated on a 4-point Likert scale (e.g., “I do not feel sad” to “I am so sad or unhappy that I can’t stand it”). Higher scores indicate higher levels of depression. The BDI has been shown to possess good psychometric properties across clinical and community samples [48] (Cronbach’s alpha of 0.82).

### 2.3. Statistical Analysis

First, a multivariate analysis of variance (MANOVA) was performed on the DERS total score, BAI scale, and BDI scale. Group (TD vs. SLD) and sex (male vs. female) were used as between-subject factors.

A second multivariate analysis of variance (MANOVA) was performed on the DERS subscales considered as dependent variables, using the group (TD vs. SLD) as a between-subject factor.

The multivariate analyses of variance (MANOVAs) were carried out using SPSS 23.0 [49] for Windows with an alpha level of 0.05.

Finally, to evaluate the mediating role of emotion regulation on the relationship between anxiety and depression mediation, analyses were computed by taking anxiety and emotional regulation as independent variables and depression as a dependent variable. The mediation analyses were conducted using the maximum likelihood estimation (MLE) method with JASP (version 0.19.3) [50]. The mediation analyses were methodologically appropriate because the three variables were associated. BAI significantly correlated with BDI (*r* = 0.720, *p* < 0.001) and DERS total score (*r* = 0.643, *p* < 0.001); DERS total scores significantly correlated with BDI (*r* = 0.766, *p* < 0.001). Since 1 out of the 3 variables did not meet the assumption of normal distribution, we performed the analysis using bias-corrected bootstrap 95% confidence intervals (1000 resamples). The analyses were performed for the entire sample, for TD subgroup and for SLD subgroup. Finally, we conducted a multigroup analysis (MGA) to test whether there were significant differences between groups in the mediation model paths [51,52].

## 3. Results

### 3.1. Differences in Emotion Regulation, Anxiety, and Depression Between SLD and TD Groups

The MANOVA revealed significant differences between both groups in terms of DERS total score (F_(1,128)_ = 10.879; *p* = 0.001; partial η2 = 0.080), with students with SLD showing significantly higher DERS total scores than TD students (SLD: mean = 93.40, SD = 22.019; TD: mean = 80.11, SD = 18.158) (see Table 2). Furthermore, the MANOVA showed a significant sex effect (sex effect: F_(1,128)_ = 4.123; *p* = 0.044; partial η2 = 0.032) and a significant sex*group interaction effect (Group*Sex interaction effect: F_(1,128)_ = 13.011; *p* < 0.001; partial η2 = 0.094). *t*-test comparisons of the DERS Total scores revealed that the females with SLD showed higher scores than the TD females (*p* < 0.001) and the SLD males (*p* = 0.001). A direct comparison of the males with SLD with the TD males did not show a difference in the DERS Total scores (*p* > 0.05).

Regarding the BAI scale (see Table 2), the MANOVA revealed a significant difference between the two groups (F_(1,128)_ = 8.060; *p* = 0.005; partial η2 = 0.061) with higher scores in students with SLD (SLD: mean = 20.24, SD = 15.376; TD: mean = 13.11, SD = 9.629). The analyses of the sex factor showed a significant difference between males and females (F_(1,128)_= 5.663; *p* = 0.019; partial η2 = 0.043), with higher scores in the female group (mean = 17.42, SD = 14.038) than in the male group (mean = 13.80, SD = 10.159). The sex*group interaction effect was significant (F_(1,128)_ = 9.962; *p* = 0.002; partial η2 = 0.074). *t*-test comparisons showed that females with SLD showed higher scores than TD females (*p* < 0.001) and SLD males (0.006). No difference was found between males in the SLD group and the TD group (*p* > 0.05).

The analyses of the depressive symptoms, i.e., the BDI scale, showed a significant difference between SLD (mean = 15.40, SD = 11.190) and TD (mean = 10.28, SD = 8.747) groups (F_(1,128)_ = 6.421; *p* = 0.013; partial η2 = 0.049) with higher scores in students with SLD (see Table 2). No significant differences between males and females (F_(1,128)_ = 0.462; *p* = 0.498; partial η2 = 0.004) were found. The sex*group interaction effect was significant (F_(1,128)_ = 6.348; *p* = 0.013; partial η2 = 0.048). *t*-test comparisons reported higher scores in the females with SLD compared to the TD females (*p* < 0.001). The comparison between SLD females and SLD males was not significant (*p* > 0.05). No difference between males with and without SLD (*p* > 0.05) emerged.

### 3.2. Differences in DERS Subtest Between SLD and TD Groups

The second MANOVA showed significant differences in three out of six DERS subscales, namely nonacceptance (F_(1,128)_ = 8.511; *p* = 0.004; partial η2 = 0.064), strategies (F_(1,128)_ = 5.171; *p* = 0.025; partial η2 = 0.040), and clarity (F_(1,128)_ = 4.205; *p* = 0.042; partial η2 = 0.033). In those scales, the comparison between groups showed higher scores in the SLD group (*p* < 0.05). For details, see Table 3.

### 3.3. Model

The analyses conducted with the mediation model support our hypothesis that emotion regulation significantly mediates the relationship between anxiety and depression (see Table 4).

**Figure 1:**
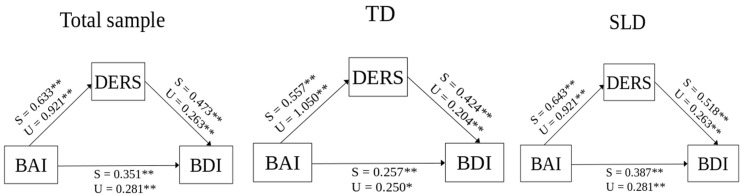
Mediation models for the entire sample and the TD and SLD subgroups. The direct effects *a* and *b* and the indirect effect *c*’ are reported. ** = *p* < 0.001; * = *p* < 0.05; S = standardized parameter; U = unstandardized parameter.

As shown in Table 4, in the total sample, TD sample, and SLD group, BAI had a positive and statistically significant direct influence on BDI (total sample: β = 0.351, *p* < 0.001; TD: β = 0.275, *p* < 0.05; SLD: β = 0.387, *p* < 0.001). In the three groups, BAI positively and significantly influenced the DERS total score (total sample: β = 0.633, *p* < 0.001; TD: β = 0.557, *p* < 0.001; SLD: β = 0.643, *p* < 0.001). Concerning the association between DERS total score and BDI, the DERS total score had a significant positive influence on BDI in the total sample (β = 0.473, *p* < 0.001), TD group (β = 0.424, *p* < 0.001) group, and SLD group (β = 0.518). As reported in Table 4, the mediation analysis (Figure 1) showed that, in the three groups, the indirect effect of BAI on BDI, mediated by DERS total score, was significant (total sample: β = 0.299, *p* < 0.001; TD: β = 0.236, *p* < 0.001; SLD: β = 0.333, *p* < 0.001). The multigroup analysis (MGA) indicated no significant differences between groups in the mediation model paths (see Table 5).

## 4. Discussion

This study aimed to investigate emotion regulation processes, anxiety, and depression in university students with SLD, comparing them with TD students. We also investigated whether specific regulatory domains are particularly affected in the SLD population. The third purpose was to examine whether or not emotion regulation moderates the relationship between anxiety and depression.

Firstly, our results indicate that students with SLD have more difficulties in emotion regulation compared to TD students. Lower emotion regulation has been recently reported by parents of Italian children with SLD [28]. Özyurt and colleagues [29] administered the DERS to adolescents with dyslexia and found more difficulties in emotion regulation compared to controls. Thus, our findings provide evidence that these emotion regulation problems also persist in early adulthood in subjects with SLD. Moreover, sex differences in DERS total score were also found, as female students reported greater emotion regulation difficulties than male students. This result is consistent with previous research showing gender-related differences in emotion regulation [53,54,55]. For instance, Malesza [55] examined the emotion regulation in a sample of 458 young adults using the DERS scale and found that females report greater general difficulties with emotion regulation than males. Overall, it has been shown that, compared to males, females use more maladaptive coping strategies [56], and feel higher levels of guilt, shame, and self-directed hostility [53]. Interestingly, we also found a significant interaction effect groups*sex in the DERS Total score. Female university students with SLD showed more difficulties in emotion regulation, relative to female controls. Thus, it would seem that being a woman and having SLD negatively enhance the influence of emotion regulation. Moreover, our data confirm the presence of higher levels of anxiety symptoms and depression in university students with SLD, compared to TD students [57,58,59]. Moreover, a sex difference was found in the anxiety scale. This data supports previous studies that described more internalizing symptoms in females than in males [60]. Interestingly, females with SLD seem to have a more compromised profile regarding emotional and psychological components [37,38,39,40,41,42,43,44,45,46,47,48,49,50,51,52,53,54,55,56,57,58,59,60,61]. Consequently, there might be an association between having SLD and being a woman as risk factors for weaker psychological health.

The second aim of this study was to identify specific regulatory domains that may be particularly affected in the SLD population. We analyzed the subscales of the DERS in more depth and found differences between SLD and TD students in three subscales, namely nonacceptance, strategies, and clarity (see Table 3). In particular, students with SLD exhibit higher scores, indicating greater difficulties in understanding and accepting stress-inducing situations, managing appropriate regulation strategies, and understanding their own emotions. Our results partially replicate those that Özyurt and colleagues [29] reported in a sample of adolescents with dyslexia. Similarly, these authors found difficulties concerning the nonacceptance of emotions. However, we did not find any significant difference on the goals and impulsivity scales. One possible explanation could be that university students may be more prone to facing and regulating emotions than adolescents. Second, in children and adolescents with SLD, the presence of deficits in executive functions (inhibition, planning, and verbal fluency) [62,63,64,65] could represent a risk factor for emotion regulation difficulties. In fact, executive functions seem to be involved in different behaviors, including problem solving, planning, and the regulation of emotion [66,67], and close relationships between emotion regulation and executive functions (i.e., attention/inhibition, working memory, and cognitive flexibility) have been also reported [68]. A different profile has been found in university students with SLD; here, working memory tended to persist as a feature of the disorder, whereas inhibition/impulse control did not differ between the TD and SLD students [69]. Interestingly, in the present study, the main difficulties found in university students concern their ability to recognize their own emotions, accept negative emotions, and regulate emotions with efficient strategies. Upon entering school, a student with SLD could potentially face a number of challenges as a result of his/her difficulties and these challenges could have a negative impact on their well-being [70]. Moreover, students with SLD tend to conceal both their negative thoughts and their academic failures to protect themselves from negative emotions [71]. Thus, university students with SLD could be less prone to focusing on how they feel and accepting negative emotions. Difficulties in facial emotion recognition have also been described in children and adolescents with SLD compared to a control group [72]. This impairment could, in turn, impact the development of social and emotional abilities. However, further investigations are needed to better understand why students with SLD show difficulties with these emotion strategies.

To our knowledge, this is the first study to investigate how emotion regulation affects the association between anxiety and depression symptoms in university students with SLD by using a mediation model, addressing our third research objective. In the present study, we confirmed the association between depression and anxiety both in the clinical sample as well as in students within the general population [73]. Moreover, we found that emotion regulation difficulties not only contribute to depressive symptoms but also have a role in the relationship between depressive symptomatology and anxiety. Emerging evidence has suggested the key role of emotion regulation in depressive symptoms in a non-clinical sample of adolescents and adults [74]. The association between emotion regulation and psychopathology was also found in clinical groups of adolescents aged between 11 and 17 years with different neuropsychiatric and neurodevelopmental disorders (ASD, ADHD, or anxiety) in which emotion dysregulation correlated and predicted both internalizing and externalizing problems [68]. Our findings are consistent with previous research highlighting the role of emotion regulation in the maintenance of psychological symptoms, as well as in the relationship between psychological variables, such as anxiety and other internalizing or externalizing symptoms [15,41,75]. Interestingly, in the present study no statistically significant differences were found between students with SLD and typically developing students regarding the associations among the variables under investigation, namely anxiety, depression, and emotion regulation. The similar association between anxiety and depression, as well as the comparable mediating role of emotion regulation observed in both groups, may be due to the fact that the students with SLD included in the study had a positive academic experience, as evidenced by their successful access to university. Moreover, students with SLD were able to use compensatory tools and, upon entering university, had access to dedicated support services for learning disorders. These resources may have served as protective factors and could help explain the similar results observed between the SLD and TD groups [36].

To our knowledge, no previous studies have investigated the mediating role of emotion regulation in university students. However, the current study emphasizes the importance of emotion regulation in psychopathological disorders and, in particular, in the maintenance of anxiety and depression. The data confirm that emotion regulation is fundamental not only for the healthy development of children and adolescents [22,23,24,25,26,27,28,29,30,31,32,33,34,35,36,37,38,39,40,41,42,43,44,45,46,47,48,49,50,51,52,53,54,55,56,57,58,59,60,61,62,63,64,65,66,67,68,69,70,71,72,73,74,75,76] but also for young adults. We claim that having adaptive emotion regulation strategies could help university students, with and without SLD, to achieve their academic goals.

### 4.1. Clinical Implications

The present study has two important clinical implications. First, the data provide guidance for the evaluation of psychological symptoms in students with SLD. Based on our results, which showed both emotion regulation difficulties in SLD students as well as the role of emotion regulation in the relationship between anxiety and depression, clinicians should pay attention to emotion regulation in subjects with SLD. Second, our findings suggest that it is important to consider emotion regulation as a potential therapeutic target of preventive and supportive intervention for university students. In addition, the data suggest the importance of monitoring emotion regulation processes in young adults, in particular those with SLD, as they can influence the expected results of psychological interventions on anxiety and depression.

### 4.2. Limitations

The results should be interpreted with consideration to some limitations. First, we only used self-completed questionnaires. Self-report biases may have contributed to errors in measurements. Moreover, measures based on questionnaires are limited in their ability to disentangle the mechanisms underlying emotion regulation, such as executive functions [77]. Given the presence of executive function difficulties in individuals with SLD [56,78,79], it could be important to consider the role of executive functions in emotion regulation processes in SLD students. Finally, studies found a strong association between inhibition difficulties and social anxiety in children [80]; thus, it could be interesting to also evaluate this specific relationship in university students with and without SLD.

### 4.3. Conclusions

In the present study, we extended the literature about emotion regulation processes and psychological symptoms in young adults and provided the first data on subjects with SLD. Our data showed that university students with SLD experience greater difficulties in emotion regulation than TD students. Specifically, our results showed that students with SLD have more difficulties in the following emotion regulation processes: nonacceptance, clarity, and strategies. Moreover, the data suggest higher levels of anxiety and more pronounced depressive symptoms in students with SLD compared to their TD peers. The study provides further evidence of the long-term psychological implications of SLD in university students. In addition, the findings highlighted that emotion regulation modulates the relationship between anxiety and depression. Therefore, our research offers novel insights into emotion regulation as a risk factor for psychopathological symptoms, including depression and anxiety, in university students with SLD.

## Figures and Tables

**Table 1:** Demographic characteristics of the SLD and the control groups.

	SLD Group (*n* = 50)	TD Group (*n* = 79)
Male	21	34
Female	29	45
Mean age (SD)	21 (2.8)	22 (2.3)

**Table 2:** Descriptive data and results of the MANOVA of the DERS total score, BAI, and BDI for the SLD and TD students.

	Group	Mean (SD)	F(df)	*p*	Partial η2
DERS Total score	TD SLD	80.11 (18.158) 93.40 (22.019)	10.879 (1)	**0.001**	0.080
BAI	TD SLD	13.11 (9.629) 20.24 (15.376)	8.060 (1)	**0.005**	0.061
BDI	TD SLD	10.28 (8.747) 15.40 (11.190)	6.421 (1)	**0.013**	0.049

*Note:* Significant results are in bold.

**Table 3:** Descriptive data and results of the MANOVA of the DERS subscales for the SLD and TD students.

	Group	Mean (SD)	F(df)	*p*	Partial η2
1. Nonacceptance	TD SLD	10.49 (4.320) 13.36 (5.450)	8.511 (1)	**0.004**	0.64
2. Goals	TD SLD	14.51 (4.966) 16.26 (4.485)	2.224 (1)	0.138	0.017
3. Strategies	TD SLD	16.84 (5.962) 19.64 (6.933)	5.171 (1)	**0.025**	0.040
4. Impulse	TD SLD	11.18 (4.904) 12.98 (5.479)	2.646 (1)	0.106	0.021
5. Clarity	TD SLD	12.20 (5.236) 14.64 (5.458)	4.2025 (1)	**0.042**	0.033
6. Awareness	TD SLD	7.51 (3.974) 8.16 (3.530)	0.701 (1)	0.404	0.006

*Note:* Significant results are in bold.

**Table 4:** Mediation analysis results for the entire sample and the SLD and TD subgroups.

Relationship	Group	Effect (LLCI-ULCI)		
BAI → DERS → BDI		Total	Direct	Indirect
	Total sample = 129	0.650 ** (0.518–0.757)	0.351 ** (0.197–0.506)	0.299 ** (0.206–0.416)
	SLD = 50	0.720 ** (0.537–0.830)	0.387 ** (0.214–0.577)	0.333 ** (0.199–0.462)
	TD = 79	0.512 ** (0.300–0.663)	0.275 * (0.020–0.492)	0.236 ** (0.110–0.405)

* *p* < 0.05; ** *p* < 0.001; LLCI = lower limit confidence interval; ULCI = upper limit confidence interval.

**Table 5:** Univariate score tests for parameter equality in the multigroup mediation analysis (BAI → DERS → BDI).

Parameter Constraint	χ^2^	df	*p*
*p*_1_ = *p*_10_	0.79	1	0.373
*p*_2_ = *p*_11_	1.35	1	0.245
*p*_3_ = *p*_12_	0.30	1	0.583

## Data Availability

The data presented in this study are available on request from the corresponding author. The data are not publicly available due to privacy reasons.
